# Establishment of the most comprehensive ITS2 barcode database to date of the traditional medicinal plant *Rhodiola* (Crassulaceae)

**DOI:** 10.1038/s41598-017-09769-y

**Published:** 2017-08-30

**Authors:** Ruo-Wei Zhu, Yuan-Cong Li, Da-Lv Zhong, Jian-Qiang Zhang

**Affiliations:** College of Life Sciences, Shaanxi Normal University, Xi’an, 710119 China

## Abstract

The roots and rhizomes of *Rhodiola crenulata* and *R. rosea* have been used worldwide as adaptogens for hundreds of years. However, rapid growth in demand has resulted in merchants using other species of *Rhodiola* as adulterants. Here, we surveyed 518 individuals representing 47 of the 55 species in the genus, including 253 *R. crenulata* individuals from 16 populations and 98 *R. rosea* individuals from 11 populations, to evaluate the utility of the internal transcribed spacer 2 (ITS2) barcode for identification of *Rhodiola* species. We detected six haplotypes in *R. crenulata* and only one haplotype in *R. rosea*. An obvious overlap between intra- and inter-specific distance was detected, and the authentication efficacy of ITS2, which was assessed by BLAST1, a nearest distance method, and a tree test, was much lower than in other groups. However, *R. crenulata* and *R. rosea* could be exactly identified. Analysis showed that the secondary structure of ITS2 differs in *R. crenulata* and its closest relatives. Our results demonstrated that both a mini barcode from ITS2 and the structure of ITS2 are effective markers for the identification of *R. crenulata* and *R. rosea*. This study represents the most comprehensive database of ITS2 barcodes in *Rhodiola* to date and will be useful in *Rhodiola* species identification.

## Introduction

DNA barcoding is a rapid, accurate means of taxonomic identification using one or a few short, standardized DNA region(s)^1,2^. After one and a half decades of effort, *CO1* was selected as the standard barcode for the identification of animal species^2^, and *rbc*L + *mat*K as core barcodes for plants^3^. The internal transcribed spacer (ITS) sequence was also recommended as a complementary marker for plant species identification^4^. However, the recommended three-barcode system has proven not to work well for the identification of plants used in traditional herbal medicine, since *rbc*L and *mat*K are very difficult to amplify from the available material, especially powdery processed products, in which DNA degradation is very common. Chen *et al*.^5^ therefore compared seven proposed candidate DNA barcodes and suggested that the internal transcribed spacer 2 (ITS2) region of nuclear ribosomal DNA would be the most suitable for barcoding medicinal plant species. Because of its high rate of successful amplification and discrimination power, ITS2 has been successfully used to identify several traditional herbal medicines in recent years^6–8^. However, successful identification should be based on a comprehensive DNA barcode database, which should include samples from all of the geographic sites of a target species’ distribution.

*Rhodiola* L. (Crassulaceae) consists of about 55 species that are mainly found at high altitudes and in cold regions of the Northern Hemisphere^9^. There are about 50 *Rhodiola* species recognized in China (16 of which are endemic), which are concentrated in the western alpine regions (i.e., the Hengduan Mountains and the Qinghai-Tibetan Plateau (QTP))^9^. Species of this genus are herbaceous perennials that often grow on gravel-covered slopes or in the cracks of exposed rocks at altitudes of approximately 3500–5000 m, making sample collection and study notoriously difficult. *Rhodiola* species, which have historically been used as adaptogens in Russia and northern Europe, are widely recognized for their ability to enhance human resistance to stress or fatigue and to promote longevity^10–12^. *Rhodiola* dietary supplements are listed as “good for handling physical and mental stress” in the consolidated list of Article 13 health claims of the European Food Safety Authority (EFSA)^13^. In China, *Rhodiola* species known as Hongjingtian have been frequently used as adaptogens, hemostatics, and tonics in traditional Tibetan medicines for hundreds of years^12^.

The roots and rhizomes of *R. crenulata* (J. D. Hooker & Thomson) H. Ohba have been included in the Pharmacopoeia of China^14^ as the authentic Rhodiolae Crenulatae Radix et Rhizoma, where they are listed as enhancing inner spiritual power, concentration, and physical endurance. This species is exclusively found in rock crevices on mountain peaks from 4300 m to 5600 m in elevation^9^, which makes it one of the highest living vascular plants in the QTP area. Since the 1980s, the accelerated and uncontrolled use of *R. crenulata* in China has severely reduced its population. Population genetic studies with inter-simple sequence repeat markers have demonstrated the low genetic diversity of this species, and it has been included in the National Class One Endangered Species in China checklist for conservation purposes^15^. The rapid increase in the commercial value of *R. crenulata* raw material has led to other *Rhodiola* species being sold as counterfeit Rhodiolae Crenulatae Radix et Rhizoma.

The type species *R. rosea* L. is a popular traditional medicinal plant in east Europe and Asia, with a reputation for stimulating the nervous system, decreasing depression, enhancing work performance, eliminating fatigue, and preventing high-altitude sickness^16^. *Rhodiola rosea* is recorded as Rhodiola rosea Root and Rhizome in the United States Pharmacopeia, and potentially confounding materials include *R. kirilowii*, *R. yunnanensis*, *R. crenulata*, *R. sacra*, and *R. sachalinensis*^17^*. Rhodiola rosea* products are sold by 46 companies worldwide, and the rapidly increasing demand for raw materials has caused *R. rosea* to become a threatened plant in many countries^18^. Thus, some substitution of other species appears in the market. In the Altay region of Russia alone, there are 24 different species of the genus which can be misclassified as *R. rosea*^19^.

A recent phylogenetic study of *Rhodiola* revealed significant convergent evolution of important morphological characters, such as dioecy and marcescent flowering stems^20^. Consequently, many of the previously defined infrageneric taxa are not monophyletic. Historical biogeographic studies have suggested that rapid radiations occurred in the evolution of this genus^21^. These evolutionary processes have made the taxonomy of *Rhodiola* notoriously difficult to evaluate. The difficulty is greater still when dealing with herbal medicines, since the officinal parts of *Rhodiola* species are roots and rhizomes, which lack diagnostic morphological characters. Thus, identification of *Rhodiola* materials based solely on morphology is problematic. DNA barcoding provides an alternative method to identify *Rhodiola* materials in the market. However, for powdery processed products, in which DNA degradation is very common, as the case in *Rhodiola* products, a shorter barcode such as ITS2 should be used. Yet we still don’t know the identification efficient of ITS2 barcode in *Rhodiola*. Besides, the successful implementation of DNA barcoding must be based on a comprehensive barcode database, which is lacking for *Rhodiola*. Our goal is thus to test the suitability of ITS2 as a barcode for identification of *Rhodiola* species, and to subsequently establish a comprehensive ITS2 barcode database for *Rhodiola* based on extensive sampling of *R. crenulata* and *R. rosea*, as well as other species in the genus.

## Results

### Amplification and sequencing

The primers for the ITS barcode were found to successfully amplify DNA from all 47 species of *Rhodiola*, and we generated 342 new ITS barcodes. We sequenced 253 *R. crenulata* individuals from 16 populations across its distribution area. We detected 6 haplotypes in these individuals. In *R. rosea*, we detected 3 haplotypes in 98 individuals. Newly generated sequences were deposited in the GenBank database with the accession numbers shown in Appendix I. In our sampling test, the sequencing success rate was 100% for the ITS sequences and 98% for the ITS2 primers.

### Sequences characteristic and barcoding gap

**Figure 1:**
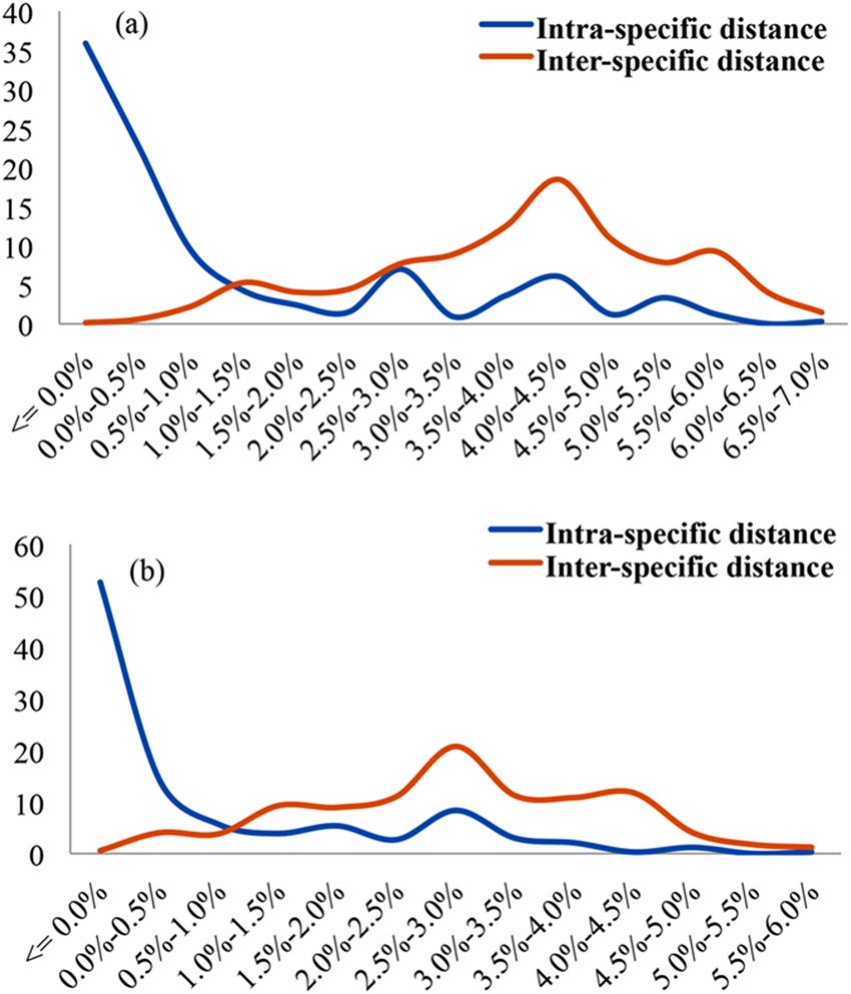
Relative distribution of inter-specific divergence between congeneric *Rhodiola* species and intra-specific variation in the ITS (**a**) and ITS2 (**b**) regions using K2P genetic distance.

The aligned length of the ITS barcode sequences was 670 bp, and the length of ITS2 barcode was 235 bp after annotation. Length variation exists in both the ITS and ITS2 regions, with a range of 600–642 bp for the former and 209–223 bp for the latter. The number of informative sites and variable sites are shown in Table 1. The original data set was analyzed to calculate the intra- and inter-specific distances. The resulting six parameters (average inter-specific distance, minimum inter-specific distance, theta prime, average intra-specific distance, coalescent depth, and theta) are shown in Table 2. The barcoding gaps between intra- and inter-specific distances are shown in Fig. 1. There was overlap between inter- and intra-specific distance in both ITS and ITS2. The median and Wilcoxon two-sample tests showed that the intra-specific distance was always significantly lower than the inter-specific distance for both ITS and ITS2 (Table 3).

**Table 1:** Sequence information for ITS and ITS2 data sets used in this study.

Data set	No. of sequences	Aligned length	Variable sites	Parsimony informative sites	Substitution model
ITS	518	670	251	201	SYM + I + G
ITS2	518	235	114	92	TVM + I

**Table 2:** Analysis of inter-specific divergence and intra-specific variation of the ITS and ITS2 sequences in samples of 47 *Rhodiola* species.

Measurement	ITS	ITS2
All interspecific distance	0.040 ± 0.015	0.028 ± 0.013
Theta prime	0.040 ± 0.016	0.028 ± 0.013
Minimum interspecific distance	0.00	0.00
All intraspecific distance	0.012 ± 0.017	0.008 ± 0.012
Theta	0.012 ± 0.017	0.008 ± 0.012
Coalescent depth	0.066	0.058

**Table 3:** Results of the median and Wilcoxon two-sample tests based on interspecific versus intraspecific Kimura 2-parameter distances for each barcode

Region	Median test	Wilcoxon two-sample test
ITS	#A = 16507 #B = 329, Median = 0.03967545, p-value < 2.2e-16	#A = 16507 #B = 329, W = 846145, p-value < 2.2e-16
ITS2	#A = 16507 #B = 329, Median = 0.02767545, p-value < 2.2e-16	#A = 16507 #B = 329, W = 4874700, p-value < 2.2e-16

#A - interspecific distances; #B - intraspecific distances; ITS, internal transcribed spacer; W, Wilcoxon two-sample test.

### The Efficacy of ITS2 for Authentication

The efficacy of the ITS and ITS2 barcodes for authentication was evaluated by both BLAST1 and the nearest distance method, as shown in Table 4. We found that 66.0% and 57.5% of the species were successfully identified as the best hit of a BLAST search using ITS and ITS2 as barcodes, respectively. The results for the nearest distance method are similar to those of the BLAST1 method. Thus, both ITS and ITS2 have a low authentication efficacy. However, in the case of *R. crenulata*, all six haplotypes could be exactly ascribed to the right species using both BLAST1 and the nearest distance method.

**Table 4:** Comparison of authentication efficiency for ITS2 using different methods.

Marker	Methods of Identification	No. of Samples	No. of Species	Correct Identification (%)	Incorrect Identification (%)	Ambiguous Identification (%)
ITS	BLAST1	518	47	31 (66.0%)	0	16 (34.0%)
	Distance	518	47	31 (66.0%)	0	16 (34.0%)
ITS2	BLAST1	518	47	27 (57.5%)	0	20 (42.5%)
	Distance	518	47	28 (59.6%)	0	19 (59.6%)

### Monophyly tests of species based on phylogenetic trees

**Figure 2:**
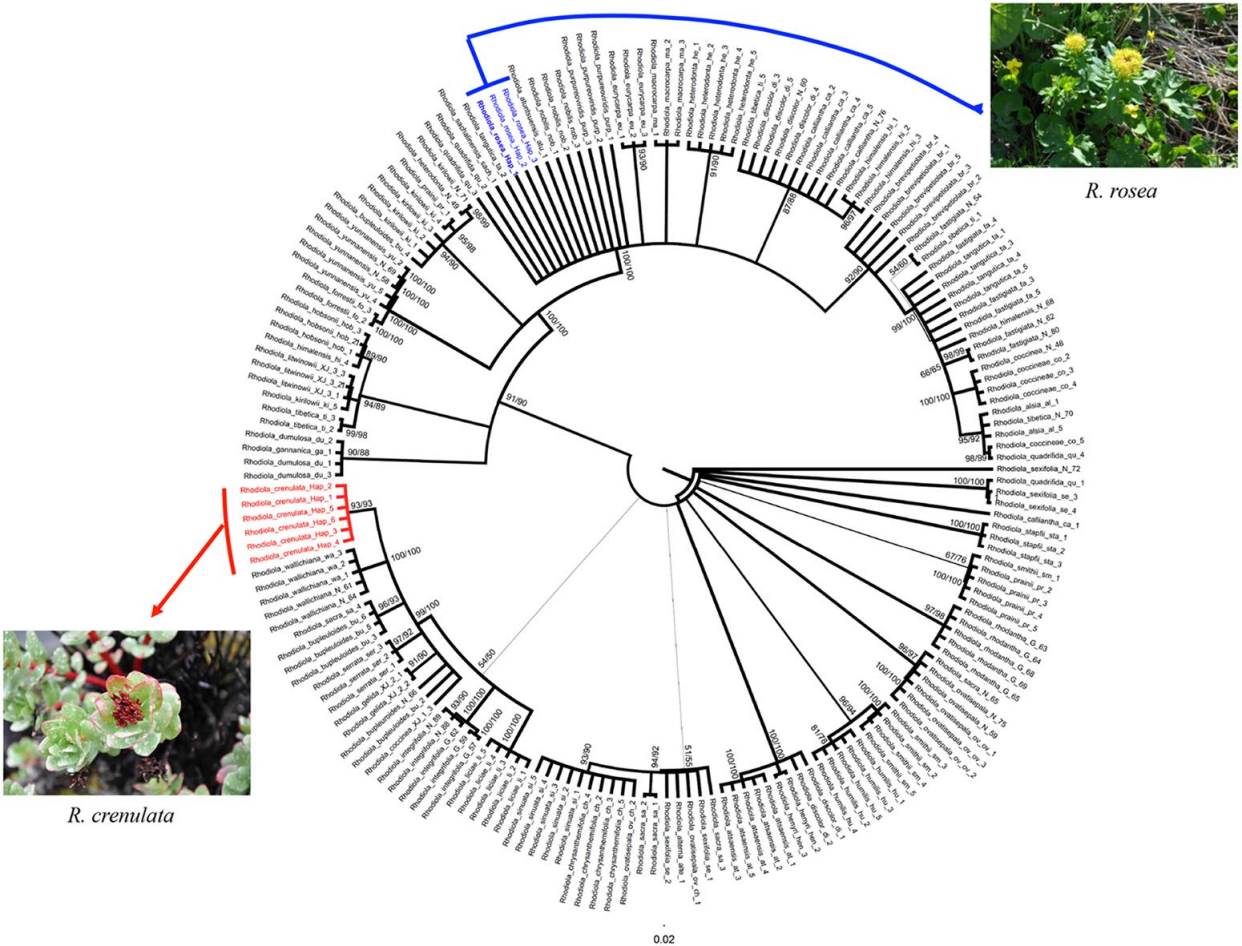
NJ tree constructed by MEGA 6 based on ITS2 sequences for *Rhodiola* species. Numbers above branches indicate bootstrap values of the NJ and maximum parsimony analyses. The width of line indicates the level of support value.

In tests of monophyly based on phylogenetic trees, the unweighted pair group method with arithmetic mean (UPGMA) analysis provided the highest discriminative power, followed by neighbor joining (NJ), and maximum parsimony (MP) analyses. ITS provided a species identification rate of 66%, while ITS2 had a success rate of 57.5%. A tree constructed by the Bayesian method based on ITS2 sequences is shown in Fig. 2. Samples from *R. crenulata* and *R. rosea* each formed a well-supported monophyletic clade.

### Analysis of SNPs

We tried to detect species- or even population-specific SNPs through SNP analysis, which has been used to identify plant species in other studies^21,22^. We used the entire data set to detect the SNPs at the interspecific level. In the ITS2 data, we found three stable variation sites at positions 141, 152 and 159 (Table 5) which can be used in combination to distinguish *R. crenulata* from other species. However, we found no stable species-specific sites identifying *R. rosea*.

**Table 5:** Three stable SNPs in ITS2 sequences from *Rhodiola crenulata* and its closely related species.

Species	SNP location		
	141 bp	152 bp	159 bp
*R. crenulata*_Hap_1	C	T	C
*R. crenulata*_Hap_2	C	T	C
*R. crenulata*_Hap_3	C	T	C
*R. crenulata*_Hap_4	C	T	C
*R. crenulata*_Hap_5	C	T	C
*R. crenulata*_Hap_6	C	T	C
*R. gelida*	T	T	C
*R. coccinea*	T	T	G

### Analysis of the Secondary Structure of the ITS2 region

**Figure 3:**
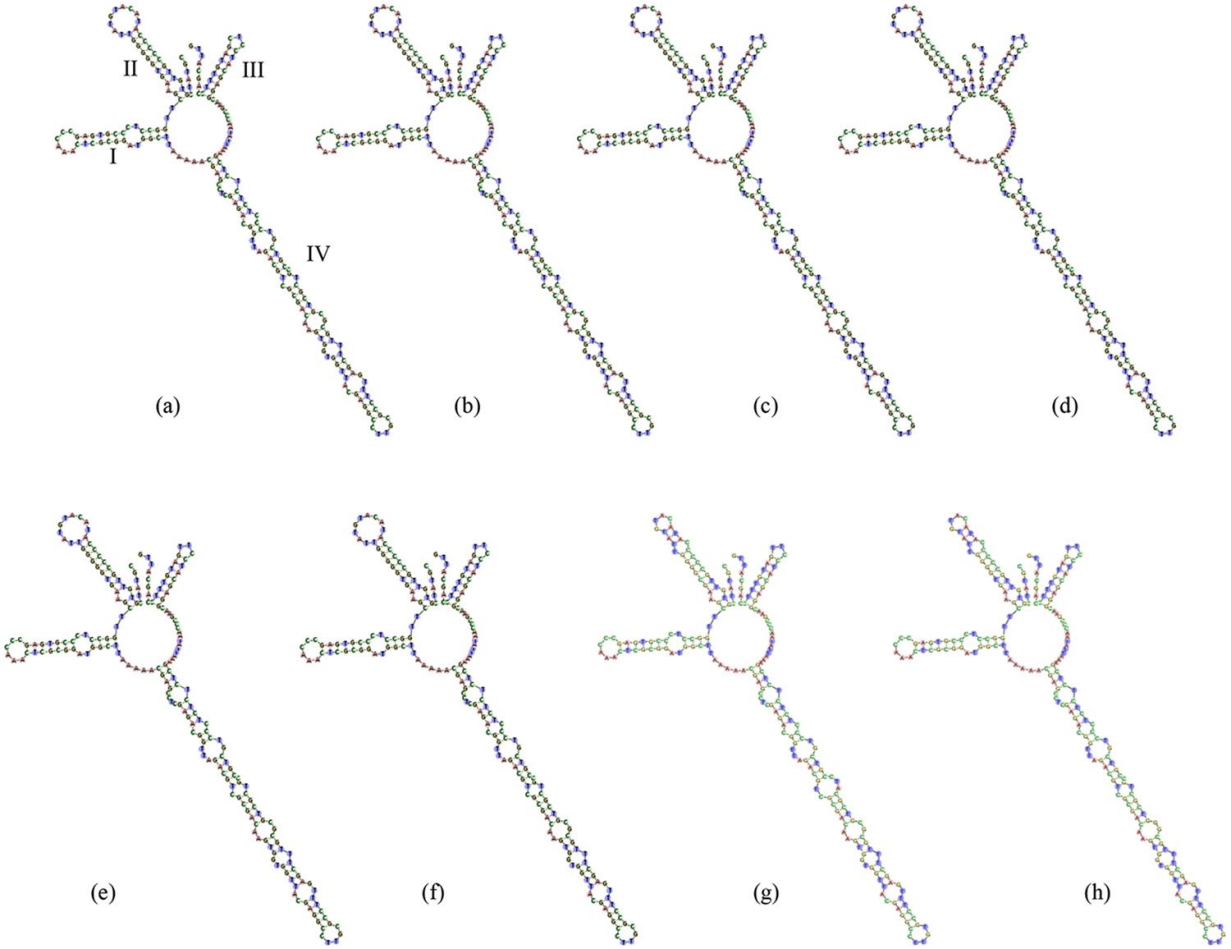
Secondary structure of ITS2 in *Rhodiola crenulata* and its adulterants. (**a-f**) six haplotypes of *R. crenulata*. (**g**) *R. gelida*; (**h**) *R. coccinea*.

**Figure 4:**
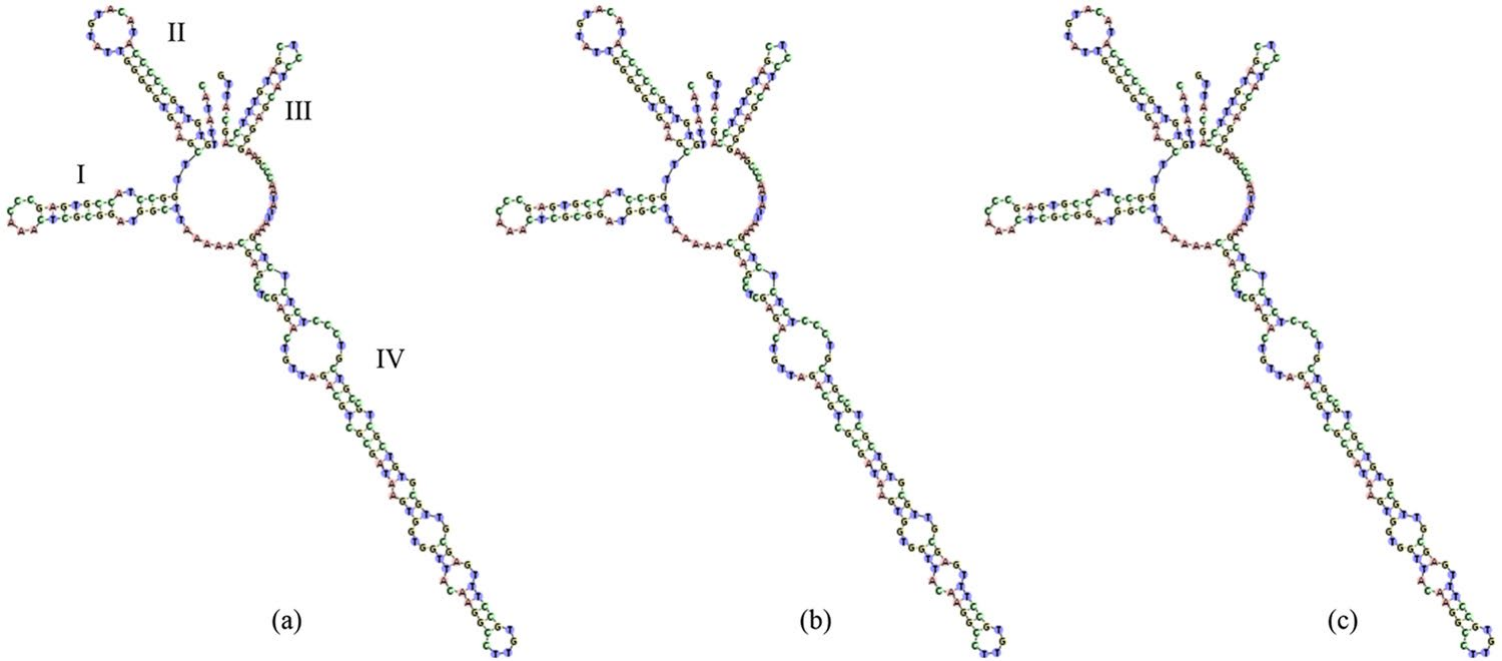
Secondary structure of ITS2 in *Rhodiola rosea* and its adulterants. (**a**) *R. rosea*; (**b**) *R. tangutica*; (**c**) *R. atuntsuensis*.

We used six haplotypes of *R. crenulata* and its closest relatives (*R. gelida* and *R. coccinea*) and one haplotype of *R. rosea* and its close relatives (*R. atuntsuensis* and *R. tangutica*) to predict the secondary structure of ITS2. All ITS2 secondary structures exhibited four similar helices: Helix I, II, III, IV (Figs 3 and 4). The secondary structure of the six *R. crenulata* haplotypes was similar but distinct from those of its close relatives in the position, size, and number of loops on Helix II and IV, which can be used as molecular morphological characteristics to identify *R. crenulata* (Fig. 3). The secondary structures of ITS2 in *R. rosea* and its related species were similar (Fig. 4).

## Discussion

DNA barcoding provides a technique that can improve descriptive taxonomy and help ensure that organisms used for various scientific, commercial, and medicinal applications are correctly identified. After more than a decade of effort, substantial progress has been made in selecting markers for barcoding. Standard markers for animals, plants, and other organisms have been established^3,5,23^. The next step for DNA barcoding is to construct complete barcode databases to assist in applications, because accurate species identification—assignment of an unknown to a known—requires a comprehensive comparative molecular database against which the unknowns can be compared.

To our knowledge, this is the first time that the ITS2 region has been used to identify *Rhodiola* species in such a large sample, an endeavor that has expanded the use of the ITS2 region to include medicinal plants. Extensive sampling and analysis of taxonomically well-understood groups is needed to thoroughly validate and standardize the markers and procedures used for DNA barcoding^24^. A partial barcode database will lead to mistakes in the identification process, especially in groups that have experienced complex evolutionary history. In the present study, we focused on two recorded medicinal plants of the genus *Rhodiola*: *R. crenulata* (Rhodiolae Crenulatae Radix et Rhizoma) and *R. rosea* (Rhodiola rosea Root and Rhizome). We sampled 253 *R. crenulata* individuals from 16 populations, covering its entire distribution area, from the Hengduan Mountains to the platform of the QTP. We also included 98 *R. rosea* individuals from 11 populations, representing samples from Asia, North America, and Europe. Only by using such a comprehensive sample to create the barcode database could we ensure that no intraspecific variation would be missed. If an incomplete database was used, future samples of *R. crenulata* or *R. rosea* would not be correctly identified, as there would be no corresponding sequences in the database.

A recent study used DNA barcoding to survey the species composition of commercial *Rhodiola* products^25^. They collected 100 Rhodiolae Crenulatae Radix et Rhizoma decoction piece samples and found that only 40% of them were authentic *R. crenulata*. However, their barcode database only included 82 voucher samples from 10 *Rhodiola* species. There are 55 species in the genus; 10 species therefore represents only 1/6 of the species diversity. Thus, a more comprehensive ITS2 barcode database is needed. In the present study, we included 47 species, covering all the morphological variation and infrageneric taxa described by previous taxonomists^9,26^. The database also includes some other medicinal *Rhodiola* species which are used by local people but are not recorded in the pharmacopeia: *R. serrate*, *R. fastigiata*, *R. himalensis*, *R. henryi*, and others. Using our database, barcoding can not only test whether a sample is authentic but also determine which species the adulterant belongs to.

It has been reported that some species of *Rhodiola* contain cyanogenic glycoside compounds, such as lotaustralin, epilotaustralin, and rhobupcyanoside A^26^. After contact with β-glucosidase and α-hydroxynitrile lyase, these compounds degrade and release hydrogen cyanide, which is harmful to humans. Species containing these compounds include *R. gelida*, *R. quadrifida*, *R. concinna*, *R. kirilowii*, *R. litwinowii*, *R. heterodonta*, and *R. bupleuroides*. Using our database, one can easily determine whether commercial products of *Rhodiola* include these species.

ITS2 is better than other candidate DNA barcodes, such as the core marker suggested for plants, *mat*K + *rbc*L, since it is more universal, has less intraspecific variation but higher interspecific divergence, and is shorter. It was therefore recommended by Chen *et al*.^5^ as a standard barcode to identify medicinal plant species. In their analysis, 90.3% of their 6600 samples could be assigned to right species using the ITS2 barcode. The efficiency of ITS2 as a barcode for medicinal plants was also evaluated in other case studies in various plant groups^7,27,28^. Our results confirm some of the previous conclusions: ITS2 is easy to amplify (98% amplification success rate), and the inter-specific genetic distances are significantly larger than intra-specific distances (Tables 2,3). However, an obvious overlap between intra- and inter-specific distance was observed (Fig. 1), and the authentication efficacy of ITS2 as assessed by BLAST1, the nearest distance method, and a tree test (Table 4 and Fig. 2) was much lower than has been reported in other studies. Zhang *et al*.^29^ showed that the core barcode combination *matK* and *rbcL* could only discriminate 40.4% of their sampled *Rhodiola* species, while ITS alone could identify 66.0% of the species, according to the phylogenetic tree test. After expanding our sample by adding 253 *R. crenulata* and 98 *R. rosea* individuals, the discrimination power of ITS was still 66%. In the ITS2 data set, phylogenetic tree analysis showed that ITS2 could discriminate 57.5% of the sampled species, less than ITS, which is not unexpected. The discrimination power of ITS2 in *Rhodiola* species is lower than in other groups^7,27^. This result is partially caused by the complex evolutionary history of the genus. *Rhodiola* species have undergone rapid radiation triggered by extensive uplift of the Qinghai-Tibetan Plateau^21^. *Rhodiola* species are difficult to identify by morphological characters, not only because their taxonomic treatment is somewhat chaotic but also because most species share similar morphology^9,26^. Using DNA barcodes to identify species has also been problematic because of the group’s complex evolutionary history, which includes interspecific hybridization, introgression, allopolyploidy, mixtures of sexual and asexual reproduction, and recent divergences^29–31^.

Although we found a lower discrimination power using the ITS2 barcode than has been reported in other studies, it is still sufficient to identify the two most widely used medicinal *Rhodiola* plants, *R. crenulata* and *R. rosea*. Since most *R. crenulata* individuals possessed the same sequences, we analyzed haplotypes of the species. In total, we detected 6 haplotypes, which formed a well-supported clade in the phylogenetic tree test (Fig. 2). The intraspecific distance ranged from 0 to 0.003. The low level of intraspecific divergence is probably due to evolutionary history: during the Pleistocene glacial, the species shrank its distribution area to the Hengduan Mountain as a refuge, then expanded very rapidly to the Qinghai-Tibetan Plateau platform after the glacial period. Due to founder effects, the genetic diversity of the populations on the QTP is very low, and we found only one haplotype in most of the populations. We tried to detect species- or even population-specific SNPs in our SNP analysis, a method which has been used to identify plant species in other studies^22,32^. Three stable sites specific to *R. crenulata* were found. These could be used as a nucleotide signature for the identification of Rhodiolae Crenulatae Radix et Rhizoma, though it should be stressed that these three sites should be used in combination. In addition, secondary structure analysis revealed differences between the haplotypes of *R. crenulata* and its closest relatives, *R. gelida* and *R. coccinea* (Fig. 3). Thus, secondary structure could be used as an alternative mini-barcode when a full-length ITS is not present. In *R. rosea*, we only detected three haplotypes in the ITS data and only one haplotype in the ITS2 data set. However, this is sufficient to correctly identify this species.

Although the ITS region was almost three times as long as the ITS2 region in the present study (670 bp *vs* 235 bp), the authentication efficacy of ITS2 assessed by BLAST1, the nearest distance method, and a tree test was not significantly lower. In practice, Chinese patent medicine samples which need to be identified are always in processed form, mostly powder or mixed with other ingredients. In these circumstances, the overall ITS region could be difficult to amplify and sequence because of DNA degradation^5^. Our results demonstrate that both a mini barcode from ITS2 and the structure of ITS2 are effective markers for the identification of *R. crenulata* and *R. rosea* based on a survey of representatives from throughout its distribution area, thus greatly broadening the application of DNA barcoding in identifying Chinese patent medicines and other products with degraded DNA.

DNA barcoding could play an important role in identifying traditional Chinese medicines and in drug safety monitoring. In addition, the use of DNA barcoding for market supervision can broaden the application of this technology and provide an excellent inspection method for supervisory institutions. In some studies, DNA barcoding has been successfully applied to supervise medicine markets. Wu *et al*.^33^ used barcoding to identify contamination of Aristolochiaceous herbs, offering a method to protect consumers from health risks caused by aristolochic acids. Zhao *et al*.^8^ used ITS2 as a barcode to identify *Acanthopanacis cortex*, and detected five adulterants in nine samples purchased from drug stores and medicine markets. In this study, we constructed a comprehensive DNA barcode database to identify *Rhodiola* products. Based on this database and a standard DNA barcoding procedure, it is possible to survey *Rhodiola* dietary supplements sold in markets. As *R. crenulata* and *R. rosea* are threatened or endangered in many countries, herbalists may substitute cheaper, more readily available species for these rare ingredients and sell them under the same name. Quality control of raw materials is key to solving this problem. An effective strategy to certify the origin of raw materials and detect adulterants in the herbal medicine markets is needed. DNA barcoding, with the support of a comprehensive barcode database, may help realize this goal by providing a means to monitor and authenticate the species found in raw materials throughout the industrial pipeline.

## Methods

### Plant Materials

We collected a total of 518 samples of *Rhodiola* species representing 47 of the 55 species in China from the provinces of Xizang, Qinghai, Gansu, Sichuan, Yunnan, and Xinjiang. We also included samples of *R. rosea* from North America and Europe (See Appendix I for collection information and NCBI accession numbers). Fresh leaves were dried in silica gel upon collection. For each taxon, three to six individuals were sampled to capture intra-specific diversity and cover most of the species’ respective geographic ranges. For species that are used for traditional medicine (i.e., *R. rosea*, and *R. crenulata*), we collected 98 and 253 individuals, respectively. These individuals represent the full range of the two species’ natural distribution area. Voucher specimens of the collected taxa were deposited in the Herbarium of Peking University (PEY) and Herbarium of Shaanxi Normal University (SANU).

### DNA extraction, amplification, and sequencing

Genomic DNA was isolated from approximated 15 mg each leaf sample following the CTAB protocol^34^. Amplification and sequencing primers were ITS-1 and ITS-4 for the ITS^35^ region (including ITS1, 5.8 S and ITS2). Polymerase chain reaction (PCR) amplification was carried out in a total volume of 20 μL containing 2 μL 10 × buffer, 0.5 μM of each primer, 200 μM of each dNTP, 1 U of *Taq* polymerase (TianGen Biotech, Beijing, China), and 1 μL template genomic DNA. The amplification followed a program of 5 min at 95 °C; 35 cycles of 1 min at 95 °C, 1 min at 56 °C, and 1 min at 72 °C; and a final extension of 5 min at 72 °C. Direct sequencing was conducted using the same primers as in the amplification. The PCR fragments were purified using polyethylene glycol (PEG) precipitation^36^. Cycle sequencing was conducted using the BigDye 3.1 reagents with an ABI 3730 automated sequencer (Applied Biosystems, Foster City, California) in Sangon Biotech Corporation (Shanghai, China). To test the utility of ITS2 primers designed by Chen *et al*.^5^, we also performed a PCR experiment in a random sample of 100 individuals without sequencing.

### Sequence analysis

Contigs were assembled and edited using ContigExpress (a component of Vector NTI Suite 6.0, InforMax). Sequences were aligned using MUSCLE 3.8.31^37^, followed by manual adjustments in Geneious 9.1^38^. The ITS2 region was delimited based on the GenBank annotation or hidden Markov models (HMMs), which were constructed through a web based tool (http://its2.bioapps.biozentrum.uni-wuerzburg.de). In the following analyses, ITS and ITS2 sequences were analyzed separately. The Kimura 2-parameter distance (K2P distance) for the two DNA regions was calculated in MEGA v. 6.0^39^ to estimate intra- and inter- specific divergence. Three parameters were used to characterize inter-specific divergence^24,40^: average inter-specific distance between all species; theta prime (i.e. the mean pairwise distance between all samples); and the smallest inter-specific distance. In addition, we used three parameters to determine intra-specific variation: average intra-specific difference between all samples collected within each species; theta, the mean pairwise distance within each species with at least two representatives; and coalescent depth, the maximum intra-specific distance. We used Wilcoxon two-sample tests and median tests (http://www.fon.hum.uva.nl/Service/Statistics.html) to assess the significance of divergence. The barcoding gaps were graphed by comparing the distribution of intra- and inter-specific divergence for each candidate barcode. We used BLAST1 and the nearest distance methods to evaluate the species authentication efficacy^5,27,41^. In the BLAST1 method, we first constructed an ITS2 database using the BLAST program (http://blast.ncbi.nlm.nih.gov/Blast.cgi), and then used all of the *Rhodiola* ITS2 sequences as query sequences to search the database. Correct identification occurred when the best BLAST hit of the query sequence was from the expected sequences, while ambiguous identification was when the best BLAST hits for a query sequence were those of several species, including the expected species, and incorrect identification was when the best BLAST hits of the query sequence did not include the expected species. In the nearest distance method, correct identification was when the hit in our database based on the smallest genetic distances was from the query species; ambiguous identification was when several hits from our database were found to have the same smallest genetic distance to the query sequence; and incorrect identification was when the hit based on the smallest genetic distance was not from the expected species.

Several tree-building methods were used to test whether individuals from the same species form a monophyletic group in phylogenetic trees based on different built using the barcodes, tree-based methods were used to display the molecular identification results. Neighbor-joining (NJ) and unweighted pair group method with arithmetic mean (UPGMA) trees were reconstructed using MEGA v. 6.0 with the K2P model^39^. PAUP v. 4.0b10^42^ was used to generate a maximum parsimony (MP) tree with a heuristic search strategy followed by random addition starting trees with tree-bisection-reconnection (TBR) branch swapping and MulTrees selected. Gaps were treated as missing data and all characters were weighted equally. Support for individual nodes was assessed using bootstrap values^43^. Parsimony bootstrap (PB) values were obtained from 1,000 replicates of heuristic searches as described above (TBR branch swapping and MulTrees selected), but with branch swapping limited to 10,000,000 rearrangements per replicate due to memory constraints. Nucleotide substitution model parameters were determined using the Akaike information criterion (AIC) in Modeltest version 3.7^44,45^.

In attempting to identify the origin region of *R. crenulata* and *R. rosea* samples, we also used the ITS2 data set to detect single-nucleotide polymorphisms (SNP). If we could find a SNP specific to a geographic region, then it could be used not only to identify the species of a sample but also where it was collected.

### Secondary structure analysis

To infer the usefulness of secondary structure of ITS2 for the identification, we chose six haplotypes representing *R. crenulata* and one haplotype representing *R. rosea*, as well as their closely related species for secondary structure prediction using the ITS2 Workbench (http://its2.bioapps.biozentrum.uni-wuerzburg.de/)^28^.

## Electronic supplementary material

Localities, voucher information and GenBank accessions numbers for sequenced taxa.
