# Author Correction: Establishment of the most comprehensive ITS2 barcode database to date of the traditional medicinal plant *Rhodiola* (Crassulaceae)

**DOI:** 10.1038/s41598-018-24988-7

**Published:** 2018-04-25

**Authors:** Ruo-Wei Zhu, Yuan-Cong Li, Da-Lv Zhong, Jian-Qiang Zhang

**Affiliations:** College of Life Sciences, Shaanxi Normal University, Xi’an, 710119 China

Correction to: *Scientific Reports* 10.1038/s41598-017-09769-y, published online 30 August 2017

The original version of this Article contained an error in the title of the paper, where the family name “Crassulaceae” was incorrectly given as “Crassulacaee”. This has now been corrected in the PDF and HTML versions of the Article, and in the accompanying Supplementary Material.

