# Supplementary material for: Establishment of the most comprehensive ITS2 barcode database to date of the traditional medicinal plant Rhodiola (Crassulaceae)
Source: Sci Rep. 2017 Aug 30;7:10051. doi: 10.1038/s41598-017-09769-y (PMC5577295; doi:10.1038/s41598-017-09769-y)
Supplement: Supplementary file 1 — Localities, voucher information and GenBank accessions numbers for sequenced taxa. [file 41598_2017_9769_MOESM1_ESM.pdf]

Establishment of the most comprehensive ITS2 barcode database to date for traditional medicinal plant *Rhodiola* (Crassulaceae)

Ruo-Wei Zhu, Yuan-Cong Li, Da-Lv Zhong, Jian-Qiang Zhang*

College of Life Sciences, Shaanxi Normal University, Xi'an 710119, China

* Email: jqzhang@snnu.edu.cn (J. Q. Zhang)

Appendix I. Localities, voucher information and GenBank accessions numbers for sequenced taxa.

Taxon	Latitude (N)	Longitude (E)	Voucher	ITS	*DNA no.*
Rhodiola alsia (Fröd.) S. H. Fu	32° 46' 30"	97° 03' 83"	*J. Q. Zhang et al. 100829-04* (PEY)	KF113683	al_1
Rhodiola alsia (Fröd.) S. H. Fu	30° 04' 45"	101° 48' 23"	*J. Q. Zhang et al. 110812-06* (PEY)	KP114694	al_5
Rhodiola alterna S. H. Fu	31° 45' 12"	94° 28' 52"	*G. Y. Rao 090803-01*(PEY)	KF113684	alte_1
Rhodiola atsaensis (Fröd.) H. Ohba	30° 04' 09"	101° 48' 08"	*J. Q. Zhang et al. 110812-04-01* (PEY)	KF113685	at_1
Rhodiola atsaensis (Fröd.) H. Ohba	30° 04' 11"	101° 48' 09"	*J. Q. Zhang et al. 110812-04-05* (PEY)	KP114695	at_2
Rhodiola atsaensis (Fröd.) H. Ohba	30° 04' 08"	101° 48' 14"	*J. Q. Zhang et al. 110812-04-03* (PEY)	KP114696	at_3
Rhodiola atsaensis (Fröd.) H. Ohba	30° 04' 06"	101° 48' 07"	*J. Q. Zhang et al. 110812-04-02* (PEY)	KP114697	at_4
Rhodiola atsaensis (Fröd.) H. Ohba	30° 04' 15"	101° 48' 05"	*J. Q. Zhang et al. 110812-04-04* (PEY)	KP114698	at_5
Rhodiola atuntsuensis (Praeger) S. H. Fu	29° 42' 22"	98° 00' 25"	*Tibet-MacArthur 2478* (US)	KJ569922	N-56|atu_1
Rhodiola brevipetiolata (Fröd.) S. H. Fu	29° 08' 21"	100° 03' 18"	*J. Q. Zhang et al. 110809-06* (PEY)	KF113686	br_1
Rhodiola brevipetiolata (Fröd.) S. H. Fu	29° 08' 13"	100° 03' 19"	*J. Q. Zhang et al. 110809-06-04* (PEY)	KP114699	br_2
Rhodiola brevipetiolata (Fröd.) S. H. Fu	29° 08' 19"	100° 03' 23"	*J. Q. Zhang et al. 110809-06-09* (PEY)	KP114700	br_3
Rhodiola brevipetiolata (Fröd.) S. H. Fu	30° 04' 14"	101° 48' 14"	*J. Q. Zhang et al. 110812-01-5c* (PEY)	KP114701	br_4
Rhodiola brevipetiolata (Fröd.) S. H. Fu	30° 04' 13"	101° 48' 12"	*J. Q. Zhang et al. 110812-01-10* (PEY)	KP114702	br_5
Rhodiola bupleuroides (Wall. ex Hook. f. & Thomson) S. H. Fu	29° 50' 18"	92° 19' 26"	*G. Y. Rao 090728-02* (PEY)	KP114703	bu_2
Rhodiola bupleuroides (Wall. ex Hook. f. & Thomson) S. H. Fu	28° 53' 38"	90° 10' 18"	*G. Y. Rao 090729-03* (PEY)	KF113687	bu_3
Rhodiola bupleuroides (Wall. ex Hook. f. & Thomson) S. H. Fu	31° 45' 12"	94° 28' 52"	*G. Y. Rao 090803-02* (PEY)	KP114704	bu_4
Rhodiola bupleuroides (Wall. ex Hook. f. & Thomson) S. H. Fu	28° 56' 20"	87° 21' 20"	*G. Y. Rao et al. 100813-03* (PEY)	KP114705	bu_5
Rhodiola bupleuroides (Wall. ex Hook. f. & Thomson) S. H. Fu	28° 56' 31"	87° 23' 19"	*G. Y. Rao et al. 100813-04* (PEY)	KP114706	bu_6
Rhodiola bupleuroides (Wall. ex Hook. f. & Thomson) S. H. Fu	30° 07' 12"	92° 09' 26"	*Tibet-MacArthur 110* (US)	KJ569923	N-66
Rhodiola calliantha (H. Ohba) H. Ohba	28° 06' 32"	85° 57' 27"	*G. Y. Rao et al. 100814-01-00* (PEY)	KP114707	ca_1
Rhodiola calliantha (H. Ohba) H. Ohba	28° 06' 39"	85° 57' 30"	*G. Y. Rao et al. 100814-01-06*(PEY)	KP114708	ca_2
Rhodiola calliantha (H. Ohba) H. Ohba	28° 06' 42"	85° 57' 31"	*G. Y. Rao et al. 100814-01-09*(PEY)	KP114709	ca_3
Rhodiola calliantha (H. Ohba) H. Ohba	28° 06' 37"	85° 57' 33"	*G. Y. Rao et al. 100814-01-03*(PEY)	KP114710	ca_4
Rhodiola calliantha (H. Ohba) H. Ohba	28° 06' 25"	85° 57' 39"	*G. Y. Rao et al. 100814-01-01*(PEY)	KF113688	ca_5

Rhodiola calliantha (H. Ohba) H. Ohba	27° 50' 37"	85° 48' 41"	*Tibet-MacArthur 750* (US)	KJ569924	N-76
Rhodiola chrysanthemifolia (H. Lév.) S. H. Fu	28° 21' 25"	99° 02' 88"	*J. Q. Zhang et al. 110803-06-03* (PEY)	KP114711	ch_2
Rhodiola chrysanthemifolia (H. Lév.) S. H. Fu	28° 21' 23"	99° 02' 81"	*J. Q. Zhang et al. 110803-06-06* (PEY)	KF113689	ch_3
Rhodiola chrysanthemifolia (H. Lév.) S. H. Fu	28° 21' 24"	99° 02' 68"	*J. Q. Zhang et al. 110803-06-01* (PEY)	KP114712	ch_4
Rhodiola chrysanthemifolia (H. Lév.) S. H. Fu	28° 21' 28"	99° 02' 92"	*J. Q. Zhang et al. 110803-06-08* (PEY)	KP114713	ch_5
Rhodiola coccinea (Royle) Boriss	43° 23' 48"	81° 02' 14"	*G. Y. Rao et al. 120829-01* (PEY)	KP114714	XJ-1-3
Rhodiola coccinea (Royle) Boriss	28° 56' 27"	87° 23' 31"	*G. Y. Rao 100813-02* (PEY)	KF113690	co_2
Rhodiola coccinea (Royle) Boriss	28° 56' 21"	87° 23' 44"	*G. Y. Rao 100813-02-02* (PEY)	KP114715	co_3
Rhodiola coccinea (Royle) Boriss	28° 56' 37"	87° 23' 32"	*G. Y. Rao 100813-02-04* (PEY)	KP114716	co_4
Rhodiola coccinea (Royle) Boriss	30° 04' 11"	101° 48' 12"	*J. Q. Zhang et al. 110812-03-08* (PEY)	KP114717	co_5
Rhodiola coccinea (Royle) Boriss	30° 31' 11"	91° 06' 25"	*Tibet-MacArthur 3417* (US)	KJ569925	N-48
Rhodiola crenulata (Hook. f. & Thomson) H. Ohba	31° 56' 02"	98° 56' 02"	*G. Y. Rao 090722-01* (PEY)	KP114718	cr_1
Rhodiola crenulata (Hook. f. & Thomson) H. Ohba	28° 57' 28"	87° 23' 37"	*G. Y. Rao et al. 100813-01* (PEY)	KF113691	cr_2
Rhodiola crenulata (Hook. f. & Thomson) H. Ohba	29° 43' 19"	98° 00' 25"	*Tibet-MacArthur 2474* (US)	KJ569926	N-57
Rhodiola crenulata (Hook. f. & Thomson) H. Ohba	29° 50' 14"	92° 19' 32"	*Tibet-MacArthur 3256* (US)	KJ569927	N-47
Rhodiola crenulata (Hook. f. & Thomson) H. Ohba	28º07'43"	99º53'51"	*zjq20160053*		
Rhodiola crenulata (Hook. f. & Thomson) H. Ohba	29º42'46"	97º57'43"	*zjq20160070*		
Rhodiola crenulata (Hook. f. & Thomson) H. Ohba	29º42'46"	97º57'43"	*zjq20160071*		
Rhodiola crenulata (Hook. f. & Thomson) H. Ohba	29º18'36"	97º00'59"	*zjq20160084*		
Rhodiola crenulata (Hook. f. & Thomson) H. Ohba	29º33'62"	94º34'58"	*zjq20160097*		
Rhodiola crenulata (Hook. f. & Thomson) H. Ohba	29º50'02"	92º20'02"	*zjq20160105*		
Rhodiola crenulata (Hook. f. & Thomson) H. Ohba	31º22'58"	93º46'32"	*zjq20160124*		
Rhodiola crenulata (Hook. f. & Thomson) H. Ohba	31º41'34"	94º55'31"	*zjq20160134*		
Rhodiola crenulata (Hook. f. & Thomson) H. Ohba	31º56'5"	98º55'43"	*zjq20160148*		
Rhodiola crenulata (Hook. f. & Thomson) H. Ohba	31° 56' 02"	98° 56' 02"	*G. Y. Rao 090722-01* (PEY)		
Rhodiola crenulata (Hook. f. & Thomson) H. Ohba	28° 57' 28"	87° 23' 37"	*J. Q. Zhang 100813-01* (PEY)		
Rhodiola crenulata (Hook. f. & Thomson) H. Ohba	28° 57' 28"	87° 23' 37"	*J. Q. Zhang 110731-04* (PEY)		

Rhodiola crenulata (Hook. f. & Thomson) H. Ohba	28° 07' 08''	99° 54' 02''	*J. Q. Zhang 110731-07* (PEY)		
Rhodiola crenulata (Hook. f. & Thomson) H. Ohba	29° 08' 23''	100° 03' 24''	*J. Q. Zhang 110809-02* (PEY)		
Rhodiola crenulata (Hook. f. & Thomson) H. Ohba	29° 33' 62''	94° 34' 42''	*J. Q. Zhang 120717-07* (PEY)		
Rhodiola crenulata (Hook. f. & Thomson) H. Ohba	31° 48' 79''	98° 34' 78''	*J. Q. Zhang 120712-03* (PEY)		
Rhodiola crenulata (Hook. f. & Thomson) H. Ohba	28° 57' 28''	87° 23' 37''	*G. Y. Rao 100813-01* (PEY)		
Rhodiola discolor (Franch.) S. H. Fu	31° 35' 31''	98° 34' 31''	*G. Y. Rao 090723-01* (PEY)	KF113692	di_1
Rhodiola discolor (Franch.) S. H. Fu	31° 35' 31''	98° 34' 31''	*G. Y. Rao 090723-02* (PEY)	KP114719	di_2
Rhodiola discolor (Franch.) S. H. Fu	28° 07' 21''	85° 58' 02''	*J. Q. Zhang 100815-03* (PEY)	KP114720	di_3
Rhodiola discolor (Franch.) S. H. Fu	28° 29' 27''	85° 14' 19''	*J. Q. Zhang 100818-03* (PEY)	KP114721	di_4
Rhodiola discolor (Franch.) S. H. Fu	28° 30' 58''	85° 13' 10''	*J. Q. Zhang 100818-04* (PEY)	KP114722	di_5
Rhodiola discolor (Franch.) S. H. Fu	28° 06' 01''	85° 59' 25''	*Tibet-MacArthur 776* (US)	KJ569928	N_60
Rhodiola dumulosa (Franch.) S. H. Fu	39° 59' 82''	115° 25' 37''	*J. Q. Zhang 100717-05* (PEY)	KF113693	du_1
Rhodiola dumulosa (Franch.) S. H. Fu	39° 59' 87''	115° 25' 34''	*J. Q. Zhang 100717-06* (PEY)	KP114723	du_2
Rhodiola dumulosa (Franch.) S. H. Fu	39° 59' 74''	115° 25' 87''	*J. Q. Zhang 100717-07* (PEY)	KP114724	du_3
Rhodiola eurycarpa (Franch.) S. H. Fu	34° 56' 42''	103° 45' 58''	*J. Q. Zhang 120705-03-10* (PEY)	KF113694	eu_1
Rhodiola eurycarpa (Franch.) S. H. Fu	34° 56' 47''	103° 45' 59''	*J. Q. Zhang 120705-03-03* (PEY)	KP114725	eu_2
Rhodiola eurycarpa (Franch.) S. H. Fu	34° 56' 43''	103° 46' 10''	*J. Q. Zhang 120705-03-12* (PEY)	KP114726	eu_3
Rhodiola fastigiata (Hook. f. & Thomson) S. H. Fu	29° 37' 58''	94° 37' 30''	*G. Y. Rao 090727-01*(PEY)	KF113695	fa_3
Rhodiola fastigiata (Hook. f. & Thomson) S. H. Fu	28° 54' 14''	85° 22' 64''	*G. Y. Rao et al. 100819-01* (PEY)	KP114727	fa_4
Rhodiola fastigiata (Hook. f. & Thomson) S. H. Fu	28° 07' 09''	99° 54' 04''	*J. Q. Zhang 110731-01* (PEY)	KP114728	fa_5
Rhodiola fastigiata (Hook. f. & Thomson) S. H. Fu	29° 42' 58''	95° 42' 01''	*Tibet-MacArthur 2697* (US)	KJ569930	N-54
Rhodiola fastigiata (Hook. f. & Thomson) S. H. Fu	29° 43' 05''	94° 42' 51''	*Tibet-MacArthur 3021* (US)	KJ569931	N-80
Rhodiola fastigiata (Hook. f. & Thomson) S. H. Fu	29° 42' 56''	94° 36' 01''	*Tibet-MacArthur 695* (US)	KJ569932	N-62
Rhodiola forrestii (Raym.-Hamet) S. H. Fu	27° 53' 58''	99° 33' 46''	*J. Q. Zhang et al. 110730-02-01*(PEY)	KF113696	fo_2
Rhodiola forrestii (Raym.-Hamet) S. H. Fu	27° 53' 40''	99° 33' 48''	*J. Q. Zhang et al. 110730-02-02*(PEY)	KP114729	fo_3
Rhodiola gelida Schrenk	43° 23' 21''	81° 02' 10''	*G. Y. Rao et al. 120829-02-05* (PEY)	KP114730	XJ-2-2
Rhodiola gannanica K. T. Fu	34° 56' 71''	102° 55' 87''	*G. Y. Rao et al. 100731-01* (PEY)	KF113697	ga_1

Rhodiola gelida Schrenk	43° 23' 21"	81° 02' 10"	*G. Y. Rao et al. 120829-02-04* (PEY)	KJ569933	XJ-2-1
Rhodiola henryi (Diels) S. H. Fu	34° 01' 50"	107° 51' 46"	*J. Q. Zhang et al. 120820-04-01* (PEY)	KF113698	hen_1
Rhodiola henryi (Diels) S. H. Fu	34° 01' 58"	107° 52' 42"	*J. Q. Zhang et al. 120820-04-02* (PEY)	KP114731	hen_2
Rhodiola henryi (Diels) S. H. Fu	34° 01' 49"	107° 52' 47"	*J. Q. Zhang et al. 120820-04-03* (PEY)	KP114732	hen_3
Rhodiola heterodonta (Hook. f. & Thomson) Boriss.	30° 04' 47"	89° 06' 25"	*G. Y. Rao et al. 100811-02* (PEY)	KF113699	he_1
Rhodiola heterodonta (Hook. f. & Thomson) Boriss.	28° 04' 17"	85° 56' 21"	*G. Y. Rao et al. 100816-01-00* (PEY)	KP114733	he_2
Rhodiola heterodonta (Hook. f. & Thomson) Boriss.	30° 04' 47"	89° 06' 25"	*G. Y. Rao et al. 100811-02-03* (PEY)	KP114734	he_3
Rhodiola heterodonta (Hook. f. & Thomson) Boriss.	30° 04' 47"	89° 06' 25"	*G. Y. Rao et al. 100811-02-05* (PEY)	KP114735	he_4
Rhodiola heterodonta (Hook. f. & Thomson) Boriss.	28° 04' 17"	85° 56' 21"	*G. Y. Rao et al. 100816-01-04* (PEY)	KP114736	he_5
Rhodiola heterodonta (Hook. f. & Thomson) Boriss.	29° 42' 26"	94° 42' 35"	*Tibet-MacArthur 3053* (US)	KJ569934	N-49
Rhodiola himalensis (D. Don) S. H. Fu	28° 08' 44"	85° 58' 31"	*G. Y. Rao et al. 100815-01* (PEY)	KF113700	hi_1
Rhodiola himalensis (D. Don) S. H. Fu	28° 07' 45"	85° 58' 25"	*G. Y. Rao et al. 100815-01-03* (PEY)	KP114737	hi_2
Rhodiola himalensis (D. Don) S. H. Fu	28° 08' 42"	85° 58' 39"	*G. Y. Rao et al. 100815-01-05* (PEY)	KP114738	hi_3
Rhodiola himalensis (D. Don) S. H. Fu	28° 08' 59"	85° 58' 42"	*G. Y. Rao et al. 100815-01-02* (PEY)	KP114739	hi_4
Rhodiola himalensis (D. Don) S. H. Fu	28° 09' 10"	99° 54' 53"	*Tibet-MacArthur 1149* (US)	KJ569935	N-68
Rhodiola hobsonii (Prain ex Hamet) S. H. Fu	29° 33' 80"	94° 34' 57"	*J. Q. Zhang et al. 120717-05* (PEY)	KF113701	hob_1
Rhodiola hobsonii (Prain ex Hamet) S. H. Fu	29° 33' 72"	94° 34' 59"	*J. Q. Zhang et al. 120717-05-03* (PEY)	KP114740	hob_2
Rhodiola hobsonii (Prain ex Hamet) S. H. Fu	29° 33' 85"	94° 34' 39"	*J. Q. Zhang et al. 120717-05-02* (PEY)	KP114741	hob_3
Rhodiola humilis (HK. f. et Thoms.) S. H. Fu	29° 41' 58"	98° 35' 31"	*J. Q. Zhang et al. 110804-03* (PEY)	KF113702	hu_1
Rhodiola humilis (HK. f. et Thoms.) S. H. Fu	29° 41' 52"	98° 35' 32"	*J. Q. Zhang et al. 110804-03-03* (PEY)	KP114742	hu_2
Rhodiola humilis (HK. f. et Thoms.) S. H. Fu	29° 41' 47"	98° 35' 36"	*J. Q. Zhang et al. 110804-03-09* (PEY)	KP114743	hu_3
Rhodiola humilis (HK. f. et Thoms.) S. H. Fu	29° 41' 42"	98° 35' 25"	*J. Q. Zhang et al. 110804-03-02* (PEY)	KP114744	hu_4
Rhodiola humilis (HK. f. et Thoms.) S. H. Fu	29° 41' 58"	98° 35' 41"	*J. Q. Zhang et al. 110804-03-04* (PEY)	KP114745	hu_5
Rhodiola integrifolia Raf.	65° 02' 36"	-144° 52' 06"	*J. E. Cantlon & W. T. Gillis 57-1657* (US)	KJ569936	N-88
Rhodiola integrifolia Raf.	66° 27' 31"	-145° 50' 19"	*H. T. Shacklette 6294* (US)	-	N-89
Rhodiola integrifolia Raf.	65° 24' 36"	-145° 59' 06"	*H. Guest 5182*(UVIC)	KF113703	G-57
Rhodiola integrifolia Raf.	64° 47' 58"	-138° 03' 49"	*H. Guest 5046* (UVIC)	KF113704	G-59

Rhodiola integrifolia Raf.	41° 21' 35"	-106° 18' 36"	*H. Guest 6025* (UVIC)	KJ569940	G-62
Rhodiola kirilowii (Regel) Maxim.	31° 43' 50"	100° 44' 09"	*G. Y. Rao 090721-01* (PEY)	KP114746	ki_1
Rhodiola kirilowii (Regel) Maxim.	31° 42' 54"	100° 44' 01"	*G. Y. Rao 090721-02* (PEY)	KP114747	ki_2
Rhodiola kirilowii (Regel) Maxim.	31° 31' 07"	96° 21' 27"	*G. Y. Rao 090806-01* (PEY)	KP114748	ki_3
Rhodiola kirilowii (Regel) Maxim.	39° 59' 34"	115° 25' 28"	*J. Q. Zhang 100717-01-RZ* (PEY)	KF113705	ki_4
Rhodiola kirilowii (Regel) Maxim.	32° 51' 19"	97° 08' 73"	*J. Q. Zhang 100829-01* (PEY)	KP114749	ki_5
Rhodiola kirilowii (Regel) Maxim.	29° 59' 51"	94° 11' 15"	*Tibet-MacArthur 3223* (US)	KJ569941	N-71
Rhodiola liciae (Raym.-Hamet) S. H. Fu	24° 56' 90"	102° 38' 37"	*J. Q. Zhang et al. 110723-01* (PEY)	KF113706	li_1
Rhodiola liciae (Raym.-Hamet) S. H. Fu	24° 55' 43"	102° 38' 32"	*J. Q. Zhang et al. 110723-01-17* (PEY)	KP114750	li_2
Rhodiola liciae (Raym.-Hamet) S. H. Fu	24° 54' 26"	102° 38' 65"	*J. Q. Zhang et al. 110723-01-18* (PEY)	KP114751	li_3
Rhodiola liciae (Raym.-Hamet) S. H. Fu	24° 56' 92"	102° 38' 24"	*J. Q. Zhang et al. 110723-01-22* (PEY)	KP114752	li_4
Rhodiola liciae (Raym.-Hamet) S. H. Fu	24° 56' 07"	102° 38' 58"	*J. Q. Zhang et al. 110723-01-07* (PEY)	KP114753	li_5
Rhodiola litwinowii Boriss.	42° 55' 78"	86° 09' 77"	*G. Y. Rao et al. 120831-01-08* (PEY)	KJ569942	XJ-3-1
Rhodiola litwinowii Boriss.	42° 55' 41"	86° 09' 72"	*G. Y. Rao et al. 120831-01-04* (PEY)	KP114754	XJ-3-2
Rhodiola litwinowii Boriss.	42° 54' 59"	86° 09' 54"	*G. Y. Rao et al. 120831-01-01* (PEY)	KP114755	XJ-3-3
Rhodiola macrocarpa (Praeger) S. H. Fu	31° 51' 80"	101° 20' 29"	*J. Q. Zhang et al. 120709-07-03* (PEY)	KF113707	macro_1
Rhodiola macrocarpa (Praeger) S. H. Fu	31° 51' 56"	101° 20' 21"	*J. Q. Zhang et al. 120709-07-01* (PEY)	KP114756	macro_2
Rhodiola macrocarpa (Praeger) S. H. Fu	31° 52' 08"	101° 20' 18"	*J. Q. Zhang et al. 120709-07-08* (PEY)	KP114757	macro_3
Rhodiola nobilis (Franch.) S. H. Fu	29° 01' 39"	92° 21' 53"	*J. Q. Zhang 120722-09-01* (PEY)	KF113708	nob_1
Rhodiola nobilis (Franch.) S. H. Fu	29° 01' 37"	92° 21' 58"	*J. Q. Zhang 120722-09-06* (PEY)	KP114758	nob_2
Rhodiola nobilis (Franch.) S. H. Fu	29° 01' 18"	92° 21' 41"	*J. Q. Zhang 120722-09-02* (PEY)	KP114759	nob_3
Rhodiola ovatisepala var. *ovatisepala* (Raym.-Hamet) S. H. Fu	28° 45' 23"	85° 32' 59"	*Tibet-MacArthur 798* (US)	-	N-75
Rhodiola ovatisepala var. *ovatisepala* (Raym.-Hamet) S. H. Fu	28° 01' 02"	85° 48' 26"	*Tibet-MacArthur 902* (US)	-	N-59
Rhodiola ovatisepala var.chingii S. H. Fu	28° 06' 80"	99° 48' 02"	*J. Q. Zhang et al. 110731-05* (PEY)	KF113710	ov_ch_1
Rhodiola ovatisepala var.chingii S. H. Fu	29° 07' 43"	100° 04' 32"	*J. Q. Zhang et al. 110809-03* (PEY)	KP114760	ov_ch_2
Rhodiola ovatisepala var. *ovatisepala* (Raym.-Hamet) S. H. Fu	28° 05' 31"	85° 57' 32"	*G. Y. Rao et al. 100814-02* (PEY)	KF113709	ov_ov_1
Rhodiola ovatisepala var. *ovatisepala* (Raym.-Hamet) S. H. Fu	28° 30' 59"	85° 13' 10"	*G. Y. Rao et al. 100818-02* (PEY)	KP114761	ov_ov_2

Rhodiola ovatisepala var. *ovatisepala* (Raym.-Hamet) S. H. Fu	28° 05' 31"	85° 57' 32"	*G. Y. Rao et al. 100814-02-02* (PEY)	KP114762	ov_ov_3
Rhodiola prainii (Raym.-Hamet) H. Ohba	28° 30' 61"	85° 13' 10"	*G. Y. Rao et al. 100818-01* (PEY)	KF113711	pr_1
Rhodiola prainii (Raym.-Hamet) H. Ohba	28° 30' 52"	85° 13' 18"	*G. Y. Rao et al. 100818-01-03* (PEY)	KP114763	pr_2
Rhodiola prainii (Raym.-Hamet) H. Ohba	28° 29' 59"	85° 13' 12"	*G. Y. Rao et al. 100818-01-04* (PEY)	KP114764	pr_3
Rhodiola prainii (Raym.-Hamet) H. Ohba	28° 30' 62"	85° 13' 29"	*G. Y. Rao et al. 100818-01-06* (PEY)	KP114765	pr_4
Rhodiola prainii (Raym.-Hamet) H. Ohba	28° 30' 66"	85° 13' 36"	*G. Y. Rao et al. 100818-01-05* (PEY)	KP114766	pr_5
Rhodiola purpureoviridis (Praeger) S. H. Fu	29° 33' 64"	94° 34' 42"	*J. Q. Zhang et al. 120717-03-09* (PEY)	KF113712	purp_1
Rhodiola purpureoviridis (Praeger) S. H. Fu	29° 33' 25"	94° 34' 41"	*J. Q. Zhang et al. 120717-03-08* (PEY)	KP114767	purp_2
Rhodiola purpureoviridis (Praeger) S. H. Fu	29° 33' 49"	94° 34' 51"	*J. Q. Zhang et al. 120717-03-15* (PEY)	KP114768	purp_3
Rhodiola quadrifida (Pall.) Fisch. et Mey.	32° 59' 68"	97° 10' 91"	*G. Y. Rao et al. 100828-01* (PEY)	KP114769	qu_1
Rhodiola quadrifida (Pall.) Fisch. et Mey.	32° 51' 18"	97° 08' 73"	*J. Q. Zhang et al. 100829-02* (PEY)	KF113714	qu_2
Rhodiola quadrifida (Pall.) Fisch. et Mey.	32° 59' 68"	97° 10' 91"	*J. Q. Zhang et al. 100828-01-1c* (PEY)	KP114770	qu_3
Rhodiola quadrifida (Pall.) Fisch. et Mey.	32° 51' 18"	97° 08' 73"	*J. Q. Zhang et al. 100829-02-2c* (PEY)	KP114771	qu_4
Rhodiola rhodantha (A. Gray) H. Jacobsen	37° 55' 43"	-107° 30' 52"	*H. Guest 6039* (UVIC)	KJ569943	G-64
Rhodiola rhodantha (A. Gray) H. Jacobsen	37° 05' 23"	-107° 29' 46"	*J. Ackerfield 3389* (CS)	KJ569944	G-68
Rhodiola rhodantha (A. Gray) H. Jacobsen	38° 02' 41"	-107° 25' 51"	*E. Hott 2664* (CS)	KJ569945	G-69
Rhodiola rhodantha (A. Gray) H. Jacobsen	40° 42' 28"	-110° 52' 07"	*G. Allen 1335* (UVIC)	-	G-65
Rhodiola rhodantha (A. Gray) H. Jacobsen	41° 21' 35"	-106° 18' 36"	*H. Guest 6026* (UVIC)	KF113715	G-63
Rhodiola rosea L.	39° 59' 21"	115° 25' 38"	*J. Q. Zhang 100717-01* (PEY)	KF113717	ro_1
Rhodiola rosea L.	39° 59' 27"	115° 25' 36"	*J. Q. Zhang 100717-02* (PEY)	KP114772	ro_4
Rhodiola rosea L.	45° 35' 38"	-64° 46' 48"	*RR2006-NB-cap* (MT)	KJ569947	G-66
Rhodiola rosea L.	66° 48' 59"	176° 11' 08"	*H. Solsad & R. Elven 05/0863* (ALA)	KJ569948	G-77
Rhodiola rosea L.	65° 02' 23"	-52° 26' 56"	*R. W. Bartlett 474* (US)	-	N-90
Rhodiola rosea L.	64° 21' 04"	-18° 08' 36"	*M. Hauksdottir s.n.* (US)	KJ569949	iceland
Rhodiola rosea L.	40° 35' 96"	117° 29' 11"	*J. Q. Zhang et al. 120616-02* (PEY)	KJ569950	N-37
Rhodiola rosea L.	39° 03' 56"	113° 38' 56"	*J. Q. Zhang et al. 120815-03* (PEY)	KJ569951	D-22
Rhodiola rosea L.	39° 03' 56"	113° 38' 56"	*J. Q. Zhang et al. 120815-03* (PEY)		

Rhodiola rosea L.	39° 02' 43"	113° 31' 50"	*J. Q. Zhang et al. 120816-05* (PEY)		
Rhodiola rosea L.	39° 57' 20"	115° 04' 08"	*J. Q. Zhang et al. 120613-01* (PEY)		
Rhodiola sachalinensis A. Bor.	42° 11' 10"	128° 10' 41"	*J. Q. Zhang 110911-02* (PEY)	KF113718	sach_1
Rhodiola sacra var. *sacra* (Prain ex Raym.-Hamet) S. H. Fu	29° 01' 22"	87° 30' 28"	*G. Y. Rao 090731-03* (PEY)	KP114773	sa_1
Rhodiola sacra var. *sacra* (Prain ex Raym.-Hamet) S. H. Fu	30° 04' 48"	89° 06' 25"	*G. Y. Rao et al. 100811-01* (PEY)	KF113719	sa_2
Rhodiola sacra var. *sacra* (Prain ex Raym.-Hamet) S. H. Fu	29° 37' 75"	91° 11' 10"	*G. Y. Rao et al. 100823-05* (PEY)	KP114774	sa_3
Rhodiola sacra var. *sacra* (Prain ex Raym.-Hamet) S. H. Fu	29° 21' 08"	90° 43' 94"	*G. Y. Rao et al. 100823-03* (PEY)	KP114775	sa_4
Rhodiola sacra var. *sacra* (Prain ex Raym.-Hamet) S. H. Fu	29° 42' 51"	91° 06' 23"	*Tibet-MacArthur 145* (US)	-	N-65
Rhodiola serrata H. Ohba	29° 06' 96"	93° 52' 29"	*J. Q. Zhang et al. 120719-04-10* (PEY)	KF113721	ser_1
Rhodiola serrata H. Ohba	29° 06' 49"	93° 52' 10"	*J. Q. Zhang et al. 120719-04-05* (PEY)	KP114776	ser_2
Rhodiola serrata H. Ohba	29° 06' 15"	93° 52' 14"	*J. Q. Zhang et al. 120719-04-03* (PEY)	KP114777	ser_3
Rhodiola sexifolia S. H. Fu	28° 07' 42"	101° 07' 79"	*J. Q. Zhang et al. 110818-01* (PEY)	KF113722	se_1
Rhodiola sexifolia S. H. Fu	30° 40' 56"	97° 15' 08"	*G. Y. Rao 090725-02* (PEY)	KP114778	se_2
Rhodiola sexifolia S. H. Fu	29° 21' 08"	90° 43' 94"	*G. Y. Rao et al. 100823-01* (PEY)	KP114779	se_3
Rhodiola sexifolia S. H. Fu	29° 21' 09"	90° 43' 52"	*G. Y. Rao et al. 100823-01-03* (PEY)	KP114780	se_4
Rhodiola sexifolia S. H. Fu	30° 02' 24"	93° 57' 02"	*Tibet-MacArthur 3135* (US)	-	N-72
Rhodiola sinuata (Royle ex Edgew.) S .H. Fu	27° 47' 61"	99° 48' 76"	*J. Q. Zhang et al. 110801-02-09* (PEY)	KF113723	si_1
Rhodiola sinuata (Royle ex Edgew.) S .H. Fu	27° 47' 66"	99° 48' 72"	*J. Q. Zhang et al. 110801-02-05* (PEY)	KP114781	si_2
Rhodiola sinuata (Royle ex Edgew.) S .H. Fu	27° 47' 63"	99° 48' 79"	*J. Q. Zhang et al. 110801-02-04* (PEY)	KP114782	si_3
Rhodiola sinuata (Royle ex Edgew.) S .H. Fu	27° 47' 50"	99° 48' 54"	*J. Q. Zhang et al. 110801-02-01* (PEY)	KP114783	si_4
Rhodiola sinuata (Royle ex Edgew.) S .H. Fu	27° 47' 48"	99° 48' 96"	*J. Q. Zhang et al. 110801-02-10* (PEY)	KP114784	si_5
Rhodiola smithii (Raym.-Hamet) S. H. Fu	29° 41' 91"	89° 05' 28"	*G. Y. Rao et al. 100810-02* (PEY)	KF113724	sm_1
Rhodiola smithii (Raym.-Hamet) S. H. Fu	28° 53' 72"	85° 22' 52"	*G. Y. Rao et al. 100819-02* (PEY)	KP114785	sm_2
Rhodiola smithii (Raym.-Hamet) S. H. Fu	29° 41' 91"	89° 05' 28"	*G. Y. Rao et al. 100810-02-06* (PEY)	KP114786	sm_3
Rhodiola smithii (Raym.-Hamet) S. H. Fu	29° 41' 91"	89° 05' 28"	*G. Y. Rao et al. 100810-02-04* (PEY)	KP114787	sm_4
Rhodiola smithii (Raym.-Hamet) S. H. Fu	28° 53' 72"	85° 22' 52"	*G. Y. Rao et al. 100819-02-02* (PEY)	KP114788	sm_5
Rhodiola stapfii (Raym.-Hamet) S. H. Fu	29° 01' 21"	92° 21' 49"	*J. Q. Zhang 120722-01-07* (PEY)	KF113726	stap_1

Rhodiola stapfii (Raym.-Hamet) S. H. Fu	29° 01' 28"	92° 21' 48"	*J. Q. Zhang 120722-01-02* (PEY)	KP114789	stap_2
Rhodiola stapfii (Raym.-Hamet) S. H. Fu	29° 01' 25"	92° 21' 59"	*J. Q. Zhang 120722-01-01* (PEY)	KP114790	stap_3
Rhodiola tangutica (Maxim.) S. H. Fu	35° 38' 55"	94° 04' 35"	*G. Y. Rao et al. 100807-01* (PEY)	KF113727	ta_1
Rhodiola tangutica (Maxim.) S. H. Fu	35° 38' 55"	94° 04' 35"	*G. Y. Rao et al. 100807-02* (PEY)	KP114791	ta_2
Rhodiola tangutica (Maxim.) S. H. Fu	35° 38' 55"	94° 04' 35"	*G. Y. Rao et al. 100807-01-4c* (PEY)	KP114792	ta_3
Rhodiola tangutica (Maxim.) S. H. Fu	35° 38' 55"	94° 04' 35"	*G. Y. Rao et al. 100807-02-7x* (PEY)	KP114793	ta_4
Rhodiola tangutica (Maxim.) S. H. Fu	35° 38' 55"	94° 04' 35"	*G. Y. Rao et al. 100807-02-10x* (PEY)	KP114794	ta_5
Rhodiola tibetica (Hook. f. & Thomson) S. H. Fu	29° 50' 18"	92° 19' 26"	*G. Y. Rao 090728-03* (PEY)	KP114795	ti_1
Rhodiola tibetica (Hook. f. & Thomson) S. H. Fu	30° 04' 46"	89° 06' 24"	*G. Y. Rao et al. 100811-03-01* (PEY)	KF113728	ti_2
Rhodiola tibetica (Hook. f. & Thomson) S. H. Fu	30° 04' 48"	89° 06' 23"	*G. Y. Rao et al. 100811-05* (PEY)	KP114796	ti_3
Rhodiola tibetica (Hook. f. & Thomson) S. H. Fu	30° 04' 49"	89° 06' 01"	*G. Y. Rao et al. 100811-05-03* (PEY)	KP114797	ti_5
Rhodiola tibetica (Hook. f. & Thomson) S. H. Fu	30° 02' 56"	94° 12' 26"	*Tibet-MacArthur 3235* (US)	-	N-70
Rhodiola wallichiana (Hook.) S. H. Fu	28° 07' 26"	85° 57' 43"	*G. Y. Rao et al. 100815-02* (PEY)	KF113730	wa_1
Rhodiola wallichiana (Hook.) S. H. Fu	28° 07' 26"	85° 57' 43"	*G. Y. Rao et al. 100815-02-01* (PEY)	KP114798	wa_2
Rhodiola wallichiana (Hook.) S. H. Fu	28° 07' 26"	85° 57' 43"	*G. Y. Rao et al. 100815-02-03* (PEY)	KP114799	wa_3
Rhodiola wallichiana (Hook.) S. H. Fu	28° 07' 25"	85° 59' 32"	*Tibet-MacArthur 472* (US)	KJ569953	N-64
Rhodiola wallichiana (Hook.) S. H. Fu	28° 05' 01"	86° 01' 20"	*Tibet-MacArthur 775* (US)	KJ569954	N-61
Rhodiola yunnanensis (Franch.) S. H. Fu	27° 53' 54"	99° 33' 42"	*J. Q. Zhang et al. 110730-01* (PEY)	KF113731	yu_2
Rhodiola yunnanensis (Franch.) S. H. Fu	27° 02' 79"	100° 11' 67"	*J. Q. Zhang et al. 110728-02-01* (PEY)	KP114800	yu_4
Rhodiola yunnanensis (Franch.) S. H. Fu	27° 02' 79"	100° 11' 67"	*J. Q. Zhang et al. 110728-02-02* (PEY)	KP114801	yu_5
Rhodiola yunnanensis (Franch.) S. H. Fu	27° 57' 21"	99° 35' 26"	*Tibet-MacArthur 2345* (US)	KJ569955	N-58
Rhodiola yunnanensis (Franch.) S. H. Fu	27° 54' 59"	99° 29' 51"	*Tibet-MacArthur 1219* (US)	KJ569956	N-69
